# Translating research into practice—implementation recommendations for pediatric rheumatology; Proceedings of the childhood arthritis and rheumatology research alliance 2020 implementation science retreat

**DOI:** 10.1186/s12969-022-00665-y

**Published:** 2022-02-07

**Authors:** Cagri Yildirim-Toruner, Rajdeep Pooni, Y. Ingrid Goh, Emily Becker-Haimes, James W. Dearing, Maria E. Fernandez, Esi M. Morgan, Gareth Parry, Jon M. Burnham, Stacy P. Ardoin, Fatima Barbar-Smiley, Joyce C. Chang, Peter Chiraseveenuprapund, Vincent Del Gaizo, Guy Eakin, Lisa C. Johnson, Yukiko Kimura, Andrea M. Knight, Melanie Kohlheim, Erica F. Lawson, Mindy S. Lo, Nancy Pan, Andrea Ring, Tova Ronis, Rebecca E. Sadun, Emily A. Smitherman, Alysha J. Taxter, Janalee Taylor, Richard K. Vehe, Sheetal S. Vora, Jennifer E. Weiss, Emily von Scheven, MAS for the CARRA Implementation Science Workgroup

**Affiliations:** 1 Baylor College of Medicine, 1102 Bates Avenue Suite 330, Houston, TX 77030 USA; 2 Stanford University School of Medicine, Lucile Packard Children’s Hospital, Stanford Children’s Health, Palo Alto, CA USA; 3 The Hospital for Sick Children, Toronto, Ontario Canada; 4 University of Pennsylvania, Perelman School of Medicine, Philadelphia, PA USA; 5 Michigan State University, Michigan, MI USA; 6 University of Texas Health Science Center at Houston, Houston, TX USA; 7 University of Washington, Seattle, WA USA; 8 Boston Children’s Hospital, (formerly at Institute for Healthcare Improvement (IHI)), Boston, MA USA; 9 Children’s Hospital of Philadelphia, Philadelphia, PA USA; 10 Nationwide Children’s Hospital, Columbus, OH USA; 11 University of California, San Diego, USA; 12 CARRA, Partnerships and Patient Engagement, Milwaukee, WI USA; 13 Arthritis Foundation, Atlanta, GA USA; 14 The Dartmouth Institute for health policy and Clinical Practice, Lebanon, NH USA; 15 The Joseph M. Sanzari Children’s Hospital, Hackensack Meridian School of Medicine, Hackensack, NJ, USA; 16 University of California, San Francisco, San Francisco, CA USA; 17 Hospital for Special Surgery, Weill Medical College of Cornell University, New York, NY, USA; 18 Children’s National Hospital, George Washington University, Washington, DC USA; 19 Duke University, Durham, NC USA; 20 University of Alabama/ Children’s of Alabama, Birmingham, AL USA; 21 Wake Forest University, Winston-Salem, NC USA; 22 Cincinnati Children’s Hospital, OH Cincinatti, USA; 23 University of Minnesota, Minnesota, MN USA; 24 Atrium Health Levine Children’s Hospital, Charlotte, NC USA

**Keywords:** Implementation sciences, Knowledge translation, Dissemination, Implementation, Strategy, Framework, Pediatric rheumatology

## Abstract

The translation of research findings into clinical practice is challenging, especially fields like in pediatric rheumatology, where the evidence base is limited, there are few clinical trials, and the conditions are rare and heterogeneous. Implementation science methodologies have been shown to reduce the research- to- practice gap in other clinical settings may have similar utility in pediatric rheumatology. This paper describes the key discussion points from the inaugural Childhood Arthritis and Rheumatology Research Alliance Implementation Science retreat held in February 2020. The aim of this report is to synthesize those findings into an Implementation Science Roadmap for pediatric rheumatology research. This roadmap is based on three foundational principles: fostering curiosity and ensuring discovery, integration of research and quality improvement, and patient-centeredness. We include six key steps anchored in the principles of implementation science. Applying this roadmap will enable researchers to evaluate the full range of research activities, from the initial clinical design and evidence acquisition to the application of those findings in pediatric rheumatology clinics and direct patient care.

## Introduction

This report summarizes the presentations and recommendations from the first Childhood Arthritis and Rheumatology Research Alliance (CARRA) Implementation Science Retreat held in Dallas, Texas, from February 24–25, 2020. The purpose of the retreat was to bring together stakeholders from across the pediatric rheumatology community to introduce the discipline of implementation science and to lay the groundwork for applying implementation science principles and methodology to pediatric rheumatic disease research. The retreat resulted in the development of pediatric rheumatology-specific implementation science roadmap and identified critical over-arching principles for how to approach this work. Together, the roadmap and principles establish a foundation to guide researchers on the conduct of implementation research projects that will align with current research findings and drive clinical practice changes for the benefit of our patients with pediatric rheumatic disease.

## Background

Medical research aims to advance science to improve health outcomes for patients. Trillions of dollars are invested annually into medical research [1] but despite this enormous investment, relatively few research results have a clinically significant impact [2]. In addition, approximately 25–50% of research results are not disseminated through peer-reviewed journals [3–5]. Even when research reveals new effective treatment approaches, it can take a long time for this information to be translated into practice and only about half of recommended healthcare practices are ever implemented. Some studies suggest that it takes an average of 17 years for 14% of research findings to be adopted into every practice using traditional approaches [6, 7]. Furthermore, translating knowledge consistently across diverse populations has proven especially difficult and likely contributes to health disparities in.

our communities since better- resourced clinical settings are more likely to adopt innovations sooner than poor-resourced clinics [8, 9]. Given that health outcomes could potentially be improved by translating research results into practice [10, 11], researchers have a responsibility to better understand the barriers and facilitators to implementation and take that knowledge into account when designing interventions for clinical settings.

The implementation of clinical research findings in pediatric rheumatology is additionally challenging due to a limited evidence base, a paucity of clinical trials involving potential therapeutics, rarity of the conditions, and disease heterogeneity. Application of implementation science methods to promote systematic uptake of research findings [12] has been shown to decrease the knowledge-to-practice gaps in several pediatric clinical areas [13], including childhood asthma [14] and pediatric cancer precision medicine [15]. However, very little implementation work has occurred in the field of pediatric rheumatology. In recognition of this need, in 2019 the CARRA Implementation Science workgroup was formed in collaboration with the Pediatric Rheumatology Care and Outcomes Improvement Network (PR-COIN), a quality improvement learning health network [16], to facilitate the implementation of evidence-based practices in pediatric rheumatology. CARRA is a member-driven collaborative research network engaged in research across the spectrum of translational research with a primary focus on practical trials and comparative effectiveness research (CER) [17]. The organization’s breadth of engagement with research, broad member base and deep engagement with providers and patients position CARRA to contribute to the advancement of implementation science within pediatric rheumatology. A Patient- Centered Outcomes Research Institute (PCORI) grant mechanism was awarded jointly to the Arthritis Foundation (AF), CARRA, and PR-COIN to foster a learning health system in pediatric rheumatology. In the spirit of collaboration, the Implementation Science Workgroup and Implementation Science Roadmap grew out of the combined efforts of these research organizations to advance pediatric rheumatology outcomes.

The CARRA Implementation Science Retreat was held in Dallas, Texas, in February 2020 to educate the pediatric rheumatology community about implementation science methods and to provide a forum to work together on shared projects. The retreat brought together diverse stakeholders including researchers, physicians, nurses, nurse practitioners, other allied health professionals, trainees, and parents of patients with childhood rheumatic diseases, affiliated with CARRA, PR-COIN, and AF. Implementation science experts were invited to serve as speakers as well as facilitators in small group breakout sessions.

In this report we present a summary of expert speaker presentations, updates on select pediatric rheumatology research projects, results of the breakout session discussions and summary of recommendations for applying implementation science tools and quality improvement methodologies pediatric rheumatology. This report also includes a description of a pediatric rheumatology-specific implementation science roadmap developed to guide the community’s work going forward.

### CARRA Implementation Science Retreat Meeting Proceedings

*Meeting goals*: the objectives of the 2020 CARRA Implementation Science retreat were to:

- Provide attendees with foundational knowledge of implementation science including theories, frameworks, and study designs, as well as to review how quality improvement (QI) and implementation science (IS) can work synergistically;
- Review select pediatric rheumatology projects at various stages of implementation readiness to translate research results to practice and develop next steps; and
- Establish a pediatric rheumatology implementation science guidance document that stakeholders could refer to when planning to implement research findings.

*Attendees*: 52 participants attended the retreat.

*Invited speaker presentations:* The following presentations by invited speakers addressed the core concepts and terminology in the field of implementation science.

#### Introduction to Implementation Science

Emily Becker-Haimes, PhD, whose body of work focuses on the implementation of mental health projects, provided an overview of the discipline of implementation science. She defined implementation science as the scientific study of strategies to promote the systematic uptake of research findings and other evidence-based practices into routine practice, to improve the quality and effectiveness of health services and public health [12]. She reviewed core concepts including how implementation science differs from quality improvement, types of implementation studies and a review of the abundance of implementation science theories, models and frameworks [18]. Implementation theories are generally designed to structure our observations to help us make sense of the world and determine what influences specific outcomes. A *model* generally is a simplification of some phenomenon; it is descriptive and may not explain how outcomes are achieved. A *framework* presents a structure or plan of how concepts or variables are connected, and describes categories, but does not provide explanations of how these concepts or variables are related.

Some examples include the Canadian Institutes of Health Research (CIHR) knowledge translation (KT) model [19], The Consolidated Framework for Implementation Research (CFIR) [20] and The Theory of Planned Behavior [21]. The theory of Organizational Readiness defines readiness as a shared psychological state [22]. Dr. Becker-Haimes explained that all implementation science studies should ideally utilize some theory, model, or framework, based on what best suits the needs of the project, but that specific guidance around how to make this selection is lacking. She concluded by emphasizing the abundance of research to practice gaps and urging the researchers in the room to identify and address these “gaps”, and not to be intimidated by the new terminology (Appendix).

#### Designing an Implementation Science project

Gareth Parry, PhD Senior Scientist leading the evaluation team at Institute for Healthcare Improvement (IHI) and chair of the IHI Scientific Symposium, discussed how to apply the core concepts of implementation science to the design of an implementation study. He emphasized the importance of pre-specifying the goals and approach beginning with “bringing the right people to the table.” Optimally the work should be conducted with a diverse stakeholder team including clinicians, patients/families, organization implementation teams and funders, as it is critical that all stakeholders are in agreement regarding the questions that are being addressed. It can also be advantageous for the design of implementation research to involve stakeholders in coproduction of desired outcomes or goals [23]. In order to maximize applied learning, he promoted the concept of “improver and evaluators as best friends”. Dr. Parry suggested developing a study plan that included the following core design components: 1) Agreeing on the goal; which can be represented by an aim statement, 2) Describing the content theory; which represent the “what” changes can be made at a local setting, and can be supported by a change package or key driver diagram, 3) Describing the execution theory; which describes “how” changes can be tested and put into place, represented as a series of if/then statements in a logic model, 4) Defining the data measurement and learning plan; which includes the measurement plan and 5) Considering the context, which describes the “Where” and critical features of the local setting. He noted that the first four components are also represented in the “Model for Improvement”, however, the inclusion of local context is not necessarily integral to local quality improvement initiatives [24].

Dr. Parry also emphasized the importance of specifying an evaluation plan that aligns with the improvement and implementation approach, and that questions of impact focus primarily on where a new approach works or can be adapted to work. He suggested considering the use of “Implementation Outcome measures” including acceptability, appropriateness, adoption, feasibility, fidelity, penetration, sustainability, and cost [25]. The questions being asked and the choice of outcomes will vary by implementation phase.

#### Diffusion of Innovation and Scaling Up

James Dearing, PhD, an expert on the diffusion of innovations, reviewed the concepts of diffusion, adoption and implementation of new evidence- based practices, programs, technologies, and policies. He defined diffusion as a “social process by which an innovation is communicated through certain channels over time among the members of a social system.” Adoption typically follows an S-shaped curve characterized by a very slow beginning, after which there is initial uptake of an innovation by “early adopters” during which adoption decisions accelerate, followed by a period of slower diffusion, as the more skeptical at last adopt.” [26] He emphasized the importance of knowing the denominator, the total number of potential adopters, when measuring adoption so that researchers can know the extent of diffusion that has taken place. Typically, early adopters represent only approximately 13.5% of the population [27]. A subset of these individuals may not be vocal advocates of an innovation, but rather serve as “social models” for others to observe or learn about. These are the informal opinion leaders who can function to trigger diffusion by others following their lead.

Several strategies, informed by behavior change science, can improve diffusion, including 1) *Providing choice*. 2) *Optimizing the attributes.* 3) *Harnessing social influence.* and 4) *Attending to timing and framing*.

#### Strategies for improving Implementation

Maria E. Fernandez, PhD, an expert on implementation mapping, discussed the concepts of organizational context, culture, and readiness to change, and their influence on implementation feasibility and success. A key point was the importance of making “the right thing to do, the easy thing to do”. She reviewed the Implementation Mapping approach [28], a method for planning implementation strategies, which follows the following five steps: 1) Conduct a needs and assets assessment and identify adopters and implementers, 2) Identify adoption and implementation outcomes, performance objectives and determinants, 3) Select theoretical methods and implementation strategies, 4) Produce implementation protocols and materials, and 5) Evaluate implementation outcomes. This approach provides a systematic approach for developing or selecting and tailoring implementation strategies, by ensuring that the strategy is informed by barriers and facilitators at the level of the individual players involved in implementation as well as a consideration of the implementation context [29].

#### Learning from PR-COIN: a quality improvement learning network

Esi Morgan, MD, as the leader of Pediatric Rheumatology Care and Outcomes Improvement Network (PR-COIN) discussed the similarities and differences between quality improvement, clinical research and implementation science and how they can work synergistically within a Learning Healthcare System (LHS) approach [30–32]. She described how PR-COIN follows Wagner’s Chronic Care Model [33, 34], and the Model for Improvement (MFI) [24], an extension of QI methodologies designed to support implementation success [35], PR-COIN uses the IHI Breakthrough Series approach to support site and participant shared learning [36] to advance quality improvement initiatives at a network level. With these approaches they have designed and conducted interventions in the areas of shared decision making [37, 38], self-management support, assessing barriers to treatment adherence and tools to overcome them [38], and a treat to target approach to care of JIA [39].

To address the topic of learning from quality improvement interventions, she cited the teaching of Lloyd Provost, PhD, and colleagues at Associates in Process Improvement [40]. Dr. Morgan reviewed five methods to design a system of learning in healthcare [41] including 1) recognition and investigation of special cause [42], 2) study of informative cases [43], 3) observational studies of the relationship between factors and responses, 4) natural experiments, and 5) and design of quality improvement with planned experimentations [41].

*Pediatric Rheumatology Project Presentations:* Investigators from five pediatric rheumatology research projects at various stages of implementation readiness presented. The presentations provided attendees with ideas for future implementation studies and insight into challenges of conducting implementation studies in pediatric rheumatology.

1. Atherosclerosis Prevention in Pediatric Lupus Erythematosus (APPLE) Trial. This was a randomized control trial (RCT) which assessed the use of statins (atorvastatin) to prevent atherosclerosis (a common complication in patients with lupus) in children with childhood onset systemic lupus erythematosus (cSLE). This team has identified a new research finding, but needs to assess its appropriateness and readiness for implementation.
2. Building a Framework to Implement Structured Transition Processes. This is a pilot study aimed at determining feasibility and acceptability of implementing a structured transition policy across 10 sites using a learning collaborative model, while simultaneously assessing the impact of these transition interventions on teens and young adults with chronic rheumatic diseases. This project is in the testing phase.
3. Implementing the CARRA Uveitis Clinical Treatment Protocols (CTPs). This is a pilot study on examining the status of the uveitis CTP at 9 CARRA Registry sites and aims to better understand the practical aspects of patient enrollment and data collection. This project is in the testing phase.
4. Treat to Target for Juvenile Idiopathic Arthritis (JIA). This project involves the pilot data from a single center quality improvement study that utilized a standardized disease activity measurement, disease activity target review, and treatment algorithms. The progress with scaling up at PR-COIN sites and barriers related to use of the treatment algorithms were presented. This project highlights the interplay between quality improvement and implementation methods in the scaling phase.
5. Start Time Optimization of Biologic Therapy in Polyarticular JIA (STOP) Study. This study aimed to compare the effectiveness of the three CARRA CTP treatment strategies (step-up, early combination, biologic only) in achieving clinical inactive disease (CID) in juvenile idiopathic arthritis (JIA) at 12 months using a prospective, observational study design [44]. They identified difficulties in aligning visits to the CTP treatment algorithms and missing visits and data as limitations. Further efforts are needed to understand barriers at the level of patient, family, provider, and insurance coverage. This study represents an implementation study in the scaling phase.

### Break-out sessions

The 90-min-long break-out sessions were structured to allow small group discussion and strategic planning for specific projects. Prior to the retreat, attendees were surveyed to identify the projects of greatest interest and those with the highest interest were selected for a breakout session. Participants were then assigned to the groups based on their preference while maintaining balance in each group. The group facilitator followed a structured discussion guide that was adapted from Maria Fernandez’s implementation mapping schema which focused on identifying facilitators and barriers. Summaries of each breakout session are provided below:

#### CARRA consensus treatment plan (CTP) Breakout

CARRA CTPs have been developed for over 10 disease states however their use has been variable across sites and over time. This group’s goal was to develop a strategy for assessing the use of CARRA CTPs and to efficiently iterate them as new data emerges, treatment options fall out of favor, and new drugs come to market. They discussed conducting surveys to assess knowledge, perceptions and barriers, strategies for leveraging the CARRA Registry to assess use of CTPs, and innovative technological solutions such as the development of an app to make the CTPs more accessible and usable. Conversations focused on testing, scaling, sustaining, and spreading.

#### Screening for Mental Health symptoms in pediatric rheumatology Breakout

This group’s goal was to develop an implementation plan for widespread screening for mental health symptoms in pediatric rheumatology clinics and connecting patients to necessary resources. This group recommended the standardized “Ask, Advise, Connect” tool as “the thing” to implement, identified the important stakeholders for implementation, and brainstormed potential barriers and implementation strategies including the development and dissemination of workflows and incentives [45].

#### Transition from Pediatric to Adult Care Breakout

This group’s goal was to develop a plan for scaling up implementation of transition services. The group decided to focus on implementation of a transition policy, building on the pilot work described above. They brainstormed the key actors, discussed the question of how much effectiveness- evidence is needed, and brainstormed strategies to collect quantitative and qualitative data on implementation outcomes.

#### CARRA Implementation Science Roadmap Breakout

This group’s goal was to develop a systematic approach for CARRA’s Implementation Science activities. Main ideas included determining priorities, guidelines for determining readiness to implement, approaches for connecting science emerging from the CARRA workgroups to the implementation initiatives, and collaboration with other organizations including PR-COIN. This group’s discussions informed the development of the recommendations below and served as the foundation for the recently launched CARRA-Arthritis Foundation Implementation Science Request for Applications (RFA) in collaboration with PR-COIN.

### Recommendations for the Implementation of pediatric rheumatology research findings

**Fig. 1:**
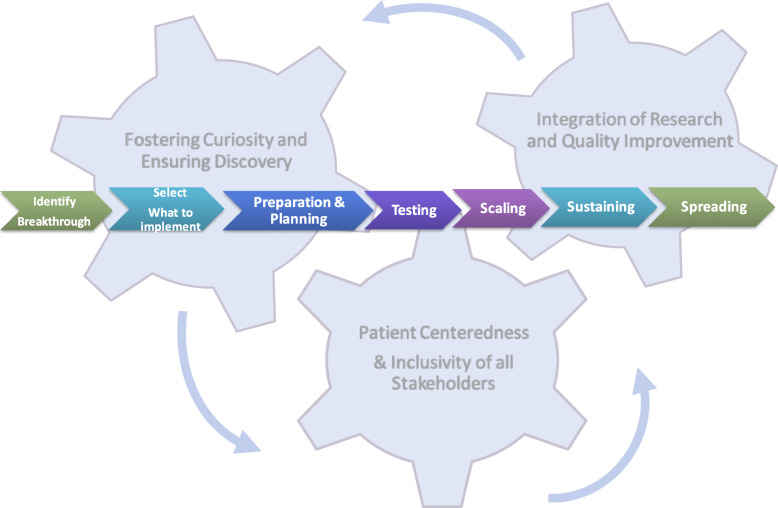
Pediatric Rheumatology Implementation Science Roadmap with overarching approaches

Following the retreat, the CARRA Implementation Science workgroup leaders synthesized the date collected from expert speakers’ presentations along with breakout group discussion notes, and identified recurrent themes to develop a six-step roadmap (Fig. 1) in order to provide a guide to researchers on the conduct of implementation science research in pediatric rheumatology. This roadmap was iteratively designed with feedback from Implementation Science workgroup members. The roadmap specifically includes tools and resources to support the investigator team. Three over-arching principles emerged as a result of the meeting, as critical to the process of bringing together the methodologies, perspectives, and values of diverse stakeholders across the fields of research and quality improvement. The three overarching principles that should be considered at each “Step” of the implementation process are as follows:

#### Fostering curiosity and ensuring discovery

CARRA’s mission is to conduct collaborative research to prevent, treat, and cure pediatric rheumatic diseases. At the core of any research initiative is the driving force of curiosity and the desire to discover new knowledge. Ensuring that implementation of new interventions achieve widespread uptake across clinical settings, in order to benefit the largest number of patients, will require continuous learning. This involves establishing reliable methods of data collection and analysis and adhering to high quality scientific methods.

#### Integration of research and quality improvement (QI)

Basic and clinical researchers and QI experts approach healthcare improvement from different paradigms using different frameworks and methods. However, they share the ultimate goal of improving patient outcomes. Envisioning the entire translational research pathway as a whole process, from pre-clinical studies, to early human studies, to clinical trials, and on through implementation and health policy enactment improves the relevance and efficiency at each step. Consideration of implementation issues during the design of translational studies and clinical trials will improve implementation potential at the end. Knowledge and understanding of early phase scientific studies will improve the design of implementation and QI initiatives. Application of QI methods to all research studies has the potential to improve the conduct of research. And application of research methods to QI studies has the potential to improve their value by also generating new knowledge. A major strength of our field is that most pediatric rheumatology researchers and QI experts also care for patients, allowing them to experience the full range of translational research through to QI and health policy in their everyday life. As a research organization, CARRA is positioned to leverage its disease-specific research committee structure to continuously evaluate the emerging evidence base to identify innovative interventions for implementation in the clinical setting. Together, utilizing its diverse membership and expertise, CARRA also has the opportunity to bring together basic and clinical researchers, QI experts, patients/families, and advocacy organizations to work collaboratively along the translational research continuum thereby accelerating the conduct of research and improvement in care and health outcomes. Together CARRA and PR-COIN activities span the spectrum from research to QI. Working collaboratively with the Arthritis Foundation, an advocacy organization with broad community engagement, these three organizations formed the CARRA Implementation Science Workgroup, organized the Implementation Science retreat, and developed the conclusions presented in this report. Continued coordination of activities within an implementation science framework will provide future opportunities to translate research findings into clinical practice in pediatric rheumatology.

#### Patient-centeredness

Inclusion of patients and parents in the planning and conduct of research allows them to contribute their unique perspectives and lived experience of illness, which improves the process and the practical impact of the research. Patients and families can be engaged at all stages of the research process, including development of research questions, study design, creation of patient-facing materials, data analysis and interpretation and dissemination of results. Patients and parents should also be included in the design, conduct and evaluation of implementation science studies. This will lead to higher relevance and better- quality studies for the benefit of those for whom they are intended, eventually improved implementation success by engaging end users in the process.

In addition to the three principles we mentioned so far, implementation science is also related to the following associated but distinct disciplines: 1) Change Management [46] 2) Project Management [47] practices that are already in place in the hospitals. Strategic business objectives that guide the alignment of projects with each organization’s overall objective should be emphasized while developing the strategic roadmap.

### Pediatric rheumatology implementation science roadmap

The following six-step roadmap was developed to guide the conduct of implementation science research in pediatric rheumatology. It charts the implementation process, beginning with the identification of a new research finding that should be translated to clinical practice, and ending with the spread of validated practices across diverse settings:

#### Step 1a: Identifying the evidence-based scientific breakthrough for Implementation

The entire scientific community should be continuously assessing scientific breakthroughs to.

identify interventions, treatments, and programs for implementation and spread. There is a responsibility to the patient community to ensure that a process is in place to support this activity. This could be achieved with a structured process embedded in existing activities, such as the American College of Rheumatology (ACR) and Paediatric Rheumatology European Society (PRES) annual meetings and other pediatric rheumatology committees including CARRA disease-specific committee reports. Alternatively, new strategies could be developed.

#### Step 1b: Selecting what will be implemented

A critical appraisal should be conducted to assess the appropriateness and potential of the selected intervention for implementation, according to its evidence, needs of the program, fit with current initiatives and supports, resource availability, readiness to implement and capacity to implement [48]. The Hexagon Tool [49] can be used to facilitate making this appraisal. Ideal innovations to implement would rate highly in each of these domains and address gaps in care that have been documented across practices. Researchers should use these implementation science tools to ensure a standardized approach in evaluating a practice of interest.

#### Step 2. Preparation and planning activities

This phase includes convening the study team, identification of the study population and clinical setting or site, defining the key players and facilitators and barriers that influence their performance of the necessary implementation tasks, selection of an implementation framework, selection of the study design and development of an evaluation plan. When defining the key players consider the individuals (actors) who are important to the implementation, what exactly they need to do, and what influences their performance (i.e., determinants) [50]. It is important to identify both potential facilitators and barriers to change when attempting to implement research findings in practice [28, 51]The BARRIERS scale is a nonspecific tool which identifies potential barriers in the implementation of research [52]. This tool was developed to identify perception of barriers by clinicians, administrators, and academics when implementing research findings. A systematic review of the BARRIERS scale concluded that although this tool has been demonstrated to have internal consistency, there is limited evidence demonstrating its validity. It is recommended that barriers assessment be conducted specifically for the context being implemented [53]. It is also important to consider facilitators of implementation – what would make it easier- what structures, processes, knowledge attitudes, etc. make it more likely or easier.

Selection of implementation science frameworks should be guided by the need of the project (see discussion above). Numerous implementation sciences frameworks exist including The Promoting Action on Research Implementation in Health Services (PARiHS), CFIR, and the Practical, Robust Implementation Sustainability Model (PRISM) [54–57].

Selection of the research approach should include consideration of whether a hybrid effectiveness-implementation study is indicated (see the implementation science subway diagram). Hybrid approaches are appropriate when there is evidence for efficacy of the practice of interest, but only partial evidence for effectiveness. The hybrid effectiveness-implementation design allows researchers to gain additional information about effectiveness while studying implementation. Once the design is selected, an evaluation plan including the selection of implementation outcome measures (acceptability, adoption, appropriateness, feasibility, fidelity, cost, penetration, sustainability) should be developed. For each measure a definition and data collection strategy should be described.

#### Step 3: Testing

Testing is important prior to scaling to identify unanticipated procedural challenges. Testing can begin at a small-scale (2–3 sites) during which implementation strategies may be developed or adapted to improve performance. Design of the implementation strategy (or strategies) is a critical step that should be informed by what was learned from the assessment of facilitators and barriers (see discussion above). During this small-scale testing phase the research design such as sampling and methods, can be adapted to maximize external validity and increase acceptability to target clinicians and other stakeholders [28]. Additionally, compelling examples of successful implementation that can be used to motivate others during the subsequent scale-up phase can be collected. An implementation protocol, materials and tools should be developed to support testing. Subsequently, testing can be extended to more sites (20 or more) to test external validity and understand feasibility across diverse settings.

#### Step 4: Scaling

Scaling, which involves successful performance of the intervention at a larger number of sites, can be supported with several strategies, such as the use of partnerships and pathways [26, 57–60]. Utilization of partnerships may involve the introduction of pilot studies done in parallel though performed in different environments. CARRA is uniquely positioned to leverage partnership with PR-COIN and community-based advocacy organizations to support implementation studies as well as clinical demonstrations that prospective adopters can visit and where they can pose questions to implementers. CARRA can also utilize the pathway approach by involving CARRA Registry sites. Whereas the testing phase includes increasing acceptability and may involve adaptations, studies at the scaling phase are oriented towards evaluation of the effectiveness of implementing the intervention and expanding coverage of the intervention in a strategic, systematic fashion.

#### Step 5: Sustaining

Sustaining change is not inevitable. Successful implementation of effective practices at a limited number of sites through short-term research-led efforts does not naturally lead to sustained adoption in the participating sites nor broader adoption at additional sites. Research to understand barriers and facilitators to routine scale-up will inform the development of effective scale-up strategies. Typically continued monitoring with feedback, and support is needed to ensure sustained and consistent performance at all sites.

#### Step 6: Spreading

Spreading refers to the adoption and implementation of improvements in pediatric rheumatology across the broader community, beyond the initial testing sites. Spread is supported by communication strategies to raise awareness, dissemination of information and sharing of technical content and tools. As a community, we have a responsibility to ensure that treatments and interventions shown to improve patient’s lives are widely implemented in all patient communities.

## Conclusion

The research community has an obligation to ensure that as new knowledge is generated, it is rapidly and efficiently translated into clinical practice, so that patients may benefit. Implementation science typically begins with identification of an evidence-based practice that is under-utilized. Next, a systematic assessment is performed focusing on what is needed to increase utilization, coupled with a formal study assessing the impact of a specific implementation strategy. The methodology of implementation science allows for a systematic approach to identifying what implementation approaches are most effective, and what changes in behavior at the level of implementers, organizations and systems are needed. The conduct of implementation research studies in pediatric rheumatic diseases is challenged by the limited evidence base, disease heterogeneity and variability in clinician practice.

The 2020 CARRA Implementation Science Retreat provided an important venue for training the research community and a foundation for working collaboratively going forward. Three overarching principles were identified to guide this work: foster curiosity and ensure discovery, be patient-centered, and integrate research with quality improvement. Additionally, the importance of change management and project management principles underlined. A six-step roadmap was developed to guide the conduct of implementation science research in pediatric rheumatology. It will be important going forward for the community to focus on capacity-building and providing ongoing training opportunities in implementation science, to work towards better integration of basic and clinical research with QI activities, and to develop strategies for sustaining, scaling, and spreading effective health care practices both within and outside the pediatric rheumatology community.

## Data Availability

Data sharing is not applicable to this article as no datasets were generated or analyzed during the current study. Materials from the meeting are available from the corresponding author on reasonable request.

## Appendix

GLOSSARY of TERMS https://carragroup.org/grants-funding/applicants/implementation-science-funding-program/glossary-of-terms

## Abbreviations

ACR: American College of Rheumatology
CARRA: Childhood Arthritis and Rheumatology Research Alliance
CER: comparative effectiveness research
CFIR: Consolidated Framework for Implementation Research
CID: Clinical inactive disease
CIHR: Canadian Institutes of Health Research
cSLE: Childhood onset systemic lupus erythematosus
CTP: Consensus treatment plan
IHI: Institute for Healthcare Improvement
JIA: Juvenile idiopathic arthritis
KT: Knowledge translation
PARiHS: The Promoting Action on Research Implementation in Health Services
PCORI: Patient- Centered Outcomes Research Institute
PR-COIN: Pediatric Rheumatology Care and Outcomes Improvement Network
PRISM: Practical, Robust Implementation Sustainability Model
QI: Quality improvement
RCT: Randomized control trial
RFA: Request for applications
STOP: Start time optimization of biologic therapy in polyarticular JIA

## References

[1] How much does your country invest in R&D? 2020. Available from: http://uis.unesco.org/apps/visualisations/research-and-development-spending/.

[2] Grant J, Buxton MJ (2018). Economic returns to medical research funding. BMJ Open.

[3] Ross JS, Tse T, Zarin DA, Xu H, Zhou L, Krumholz HM (2012). Publication of NIH funded trials registered in ClinicalTrials.gov: cross sectional analysis. BMJ.

[4] Ross JS, Mulvey GK, Hines EM, Nissen SE, Krumholz HM (2009). Trial Publication after Registration in ClinicalTrials.Gov: A Cross-Sectional Analysis. PLoS Med.

[5] Dwan K, Altman DG, Arnaiz JA, Bloom J, Chan A-W, Cronin E, Decullier E, Easterbrook PJ, von Elm E, Gamble C, Ghersi D, Ioannidis JP, Simes J, Williamson PR (2008). Systematic Review of the Empirical Evidence of Study Publication Bias and Outcome Reporting Bias. PLoS ONE.

[6] Green LW, Ottoson JM, García C, Hiatt RA (2009). Diffusion Theory and Knowledge Dissemination, Utilization, and Integration in Public Health. Annu Rev Public Health.

[7] Morris ZS, Wooding S, Grant J (2011). The answer is 17 years, what is the question: Understanding time lags in translational research. J R Soc Med.

[8] McGlynn EA, Kerr EA, Adams J, Keesey J, Asch SM (2003). Quality of Health Care for Women: A demonstration of the quality assessment tools system. Med Care.

[9] McGlynn EA, Asch SM, Adams J, Keesey J, Hicks J, DeCristofaro A (2003). The quality of health care delivered to adults in the United States. N Engl J Med.

[10] Institute of Medicine (US) Committee on Quality of Health Care in America (2001). Crossing the Quality Chasm: A New Health System for the 21st Century.

[11] Berwick DM (2003). Disseminating innovations in health care. JAMA..

[12] Eccles MP, Mittman BS (2006). Welcome to Implementation Science. Implementation Sci.

[13] Wittmeier KDM, Klassen TP, Sibley KM (2015). Implementation Science in Pediatric Health Care: Advances and opportunities. JAMA Pediatr.

[14] Reeves K, O’Hare K, Shade L, Ludden T, McWilliams A, Manning M (2020). Evaluation of a shared decision-making intervention for pediatric patients with asthma in the emergency department. Implementation Sci Commun.

[15] Rapport F, Smith J, O’Brien TA, Tyrrell VJ, Mould EV, Long JC (2020). Development of an implementation and evaluation strategy for the Australian ‘Zero Childhood Cancer’ (Zero) Program: a study protocol. BMJ Open.

[16] Our Network. PR-COIN. 2020. Available from: https://www.pr-coin.org/about-network.

[17] Mellins ED, Rider LG (2007). Clinical research networks: a step towards evidence-based practice in pediatric rheumatology. Nat Rev Rheumatol.

[18] Lane-Fall MB, Curran GM, Beidas RS (2019). Scoping implementation science for the beginner: locating yourself on the “subway line” of translational research. BMC Med Res Methodol.

[19] McLean RKD, Graham ID, Bosompra K, Choudhry Y, Coen SE, MacLeod M (2012). Understanding the performance and impact of public knowledge translation funding interventions: Protocol for an evaluation of Canadian Institutes of Health Research knowledge translation funding programs. Implementation Sci.

[20] Damschroder LJ, Aron DC, Keith RE, Kirsh SR, Alexander JA, Lowery JC (2009). Fostering implementation of health services research findings into practice: a consolidated framework for advancing implementation science. Implementation Sci.

[21] Ajzen I, Kuhl J, Beckman J (1985). From intentions to actions: a theory of planned behavior. Action-control: from cognition to behavior.

[22] Weiner BJ (2009). A theory of organizational readiness for change. Implementation Sci.

[23] Elwyn G, Nelson E, Hager A, Price A (2020). Coproduction: when users define quality. BMJ Qual Saf.

[24] The Improvement Guide: A Practical Approach to Enhancing Organizational Performance. 2nd Edition. IHI - Institute for Healthcare Improvement. Available from: http://www.ihi.org:80/resources/Pages/Publications/ImprovementGuidePracticalApproachEnhancingOrganizationalPerformance.aspx.

[25] Proctor E, Silmere H, Raghavan R, Hovmand P, Aarons G, Bunger A, Griffey R, Hensley M (2011). Outcomes for Implementation research: conceptual distinctions, measurement challenges, and research agenda. Adm Policy Ment Health.

[26] Dearing JW, Cox JG (2018). Diffusion of innovations Theory, principles, and practice. Health Aff (Millwood)..

[27] Rogers EM (2003). Diffusion of innovations.

[28] Fernandez ME, ten Hoor GA, van Lieshout S, Rodriguez SA, Beidas RS, Parcel G (2019). Implementation Mapping: Using Intervention Mapping to Develop Implementation Strategies. Front Public Health.

[29] Powell BJ, Fernandez ME, Williams NJ, Aarons GA, Beidas RS, Lewis CC (2019). Enhancing the Impact of Implementation Strategies in Healthcare: A Research Agenda. Front Public Health.

[30] Britto MT, Fuller SC, Kaplan HC, Kotagal U, Lannon C, Margolis PA, Muething SE, Schoettker PJ, Seid M (2018). Using a network organisational architecture to support the development of learning healthcare systems. BMJ Qual Saf.

[31] The Ethics of Using QI Methods to Improve Health Care Quality & Safety. The Hastings Center. 2021. Available from: https://www.thehastingscenter.org/publications-resources/special-reports-2/the-ethics-of-using-qi-methods-to-improve-health-care-quality-safety/. 10.1353/hcr.2006.0054 16898359

[32] Olsen L, Aisner D, JM MG, Institute of Medicine (US) Roundtable on Evidence-Based Medicine (2007). The Learning Healthcare System: Workshop Summary.

[33] Wagner EH, Davis C, Schaefer J, Von Korff M, Austin B (1999). A survey of leading chronic disease management programs: are they consistent with the literature?. Manag Care Q.

[34] Wagner EH, Austin BT, Davis C, Hindmarsh M, Schaefer J, Bonomi A (2001). Improving chronic illness care: translating evidence into action. Health Aff (Millwood).

[35] Gerring J (2004). What Is a Case Study and What Is It Good for?. Am Polit Sci Rev.

[36] The Breakthrough Series: IHI’s Collaborative Model for Achieving Breakthrough Improvement. IHI - Institute for Healthcare Improvement. 2021. Available from: http://www.ihi.org/resources/Pages/IHIWhitePapers/TheBreakthroughSeriesIHIsCollaborativeModelforAchievingBreakthroughImprovement.aspx.

[37] Lipstein EA, Brinkman WB, Sage J, Lannon CM, DeWitt EM (2013). Understanding treatment decision making in juvenile idiopathic arthritis: a qualitative assessment. Pediatr Rheumatol Online J.

[38] Brinkman WB, Lipstein EA, Taylor J, Schoettker PJ, Naylor K, Jones K (2017). Design and implementation of a decision aid for juvenile idiopathic arthritis medication choices. Pediatr Rheumatol Online J.

[39] Favier LA, Taylor J, Rich KL, Jones KB, Vora SS, Harris JG (2018). Barriers to Adherence in Juvenile Idiopathic Arthritis: A multicenter collaborative experience and preliminary results. J Rheumatol.

[40] Designing and Testing Treat to Target as a New Care Model in JIA Across a Network of Pediatric Rheumatology Centers. ACR Meeting Abstracts. 2021. Available from: https://acrabstracts.org/abstract/designing-and-testing-treat-to-target-as-a-new-care-model-in-jia-across-a-network-of-pediatric-rheumatology-centers/.

[41] API - Associates in Process Improvement - Lloyd Provost. 2021. Available from: http://www.apiweb.org/index.php/associates/lloyd-provost.

[42] Moen RD, Nolan TW, Provost LP (2012). Quality improvement through planned experimentation.

[43] Provost LP, Murray SK (2011). The health care data guide: learning from data for improvement.

[44] Ramaswamy R, Johnson J, Hirshhorn L, Johnson JK, Sollecito WA (2020). The intersection between quality improvement and Implementation science. McLaughlin and Kaluzny’s continuous quality improvement in health care.

[45] Hackensack Meridian Health. Start Time Optimization of Biologics in Polyarticular JIA [Internet]. clinicaltrials.gov; 2019. ClinicalTrials.gov Identifier: NCT02593006. Available from: https://clinicaltrials.gov/ct2/show/NCT02593006.

[46] Becker-Haimes EM, Tabachnick AR, Last BS, Stewart RE, Hasan-Granier A, Beidas RS (2020). Evidence Base Update for Brief, Free, and Accessible Youth Mental Health Measures. J Clin Child Adolesc Psychol.

[47] Leading Change. John P. Kotter, 2012, ISBN 13-978-1-4221-8643-5.

[48] The DNA of Strategy Execution. Jack Duggal, 2019, ISBN 978-1-119-27801-6.

[49] Curran GM (2020). Implementation science made too simple: a teaching tool. Implementation Sci Commun.

[50] The Hexagon: An Exploration Tool. NIRN. 2021. Available from: https://nirn.fpg.unc.edu/resources/hexagon-exploration-tool

[51] Shaw B, Cheater F, Baker R, Gillies C, Hearnshaw H, Flottorp S (2005). Tailored interventions to overcome identified barriers to change: effects on professional practice and health care outcomes. Cochrane Database Syst Rev.

[52] Bosch M, van der Weijden T, Wensing M, Grol R (2007). Tailoring quality improvement interventions to identified barriers: a multiple case analysis. J Eval Clin Pract.

[53] BARRIERS: the barriers to research utilization scale - PubMed. 2020. Available from: https://pubmed.ncbi.nlm.nih.gov/1741634/.

[54] Kajermo KN, Boström A-M, Thompson DS, Hutchinson AM, Estabrooks CA, Wallin L (2010). The BARRIERS scale -- the barriers to research utilization scale: A systematic review. Implementation Sci.

[55] Kitson A (1997). Using evidence to demonstrate the value of nursing. Nurs Stand.

[56] Rycroft-Malone J (2004). The PARIHS framework--a framework for guiding the implementation of evidence-based practice. J Nurs Care Qual.

[57] Toolkit Part 1: Implementation Science Methodologies and Frameworks - Fogarty International Center @ NIH. Fogarty International Center. 2021. Available from: https://www.fic.nih.gov:443/About/center-global-health-studies/neuroscience-implementation-toolkit/Pages/methodologies-frameworks.aspx.

[58] Larson RS, Dearing JW., & Backer TE. (2017). Strategies to scale up social programs: pathways, Partnerships and Fidelity. Retrieved from The Wallace Foundation website: http://www.wallacefoundation.org/knowledge-center/Documents/Strategies-to-Scale-Up-Social-Programs.pdf

[59] Powell BJ, Waltz TJ, Chinman MJ, Damschroder LJ, Smith JL, Matthieu MM (2015). A refined compilation of implementation strategies: results from the Expert Recommendations for Implementing Change (ERIC) project. Implementation Sci.

[60] Simmons R, Fajans P, Ghiron L (2007). Scaling up health service delivery: from pilot innovations to policies and programmes.

